# Should Controls With Respiratory Symptoms Be Excluded From Case-Control Studies of Pneumonia Etiology? Reflections From the PERCH Study

**DOI:** 10.1093/cid/cix076

**Published:** 2017-05-27

**Authors:** Melissa M. Higdon, Laura L. Hammitt, Maria Deloria Knoll, Henry C. Baggett, W. Abdullah Brooks, Stephen R. C. Howie, Karen L. Kotloff, Orin S. Levine, Shabir A. Madhi, David R. Murdoch, J. Anthony G. Scott, Donald M. Thea, Amanda J. Driscoll, Ruth A. Karron, Daniel E. Park, Christine Prosperi, Scott L. Zeger, Katherine L. O’Brien, Daniel R. Feikin, for the PERCH Study Group, Katherine L. O’Brien, Orin S. Levine, Maria Deloria Knoll, Daniel R. Feikin, Andrea N. DeLuca, Amanda J. Driscoll, Wei Fu, Laura L. Hammitt, Melissa M. Higdon, E. Wangeci Kagucia, Ruth A. Karron, Mengying Li, Daniel E. Park, Christine Prosperi, Zhenke Wu, Scott L. Zeger, Nora L. Watson, Jane Crawley, David R. Murdoch, W. Abdullah Brooks, Hubert P. Endtz, Khalequ Zaman, Doli Goswami, Lokman Hossain, Yasmin Jahan, Hasan Ashraf, Stephen R. C. Howie, Bernard E. Ebruke, Martin Antonio, Jessica McLellan, Eunice Machuka, Arifin Shamsul, Syed M.A. Zaman, Grant Mackenzie, J. Anthony G. Scott, Juliet O. Awori, Susan C. Morpeth, Alice Kamau, Sidi Kazungu, Karen L. Kotloff, Milagritos D. Tapia, Samba O. Sow, Mamadou Sylla, Boubou Tamboura, Uma Onwuchekwa, Nana Kourouma, Aliou Toure, Shabir A. Madhi, David P. Moore, Peter V. Adrian, Vicky L. Baillie, Locadiah Kuwanda, Azwifarwi Mudau, Michelle J. Groome, Henry C. Baggett, Somsak Thamthitiwat, Susan A. Maloney, Charatdao Bunthi, Julia Rhodes, Pongpun Sawatwong, Pasakorn Akarasewi, Donald M. Thea, Lawrence Mwananyanda, James Chipeta, Phil Seidenberg, James Mwansa, Somwe wa Somwe, Geoffrey Kwenda

**Affiliations:** 1 Department of International Health, International Vaccine Access Center, Johns Hopkins Bloomberg School of Public Health, Baltimore, Maryland;; 2 Kenya Medical Research Institute–Wellcome Trust Research Programme, Kilifi;; 3 Global Disease Detection Center, Thailand Ministry of Public Health–US Centers for Disease Control and Prevention Collaboration, Nonthaburi;; 4 Division of Global Health Protection, Center for Global Health, Centers for Disease Control and Prevention, Atlanta, Georgia;; 5 International Centre for Diarrhoeal Disease Research, Bangladesh (icddr,b), Dhaka and Matlab;; 6 Department of International Health, Johns Hopkins Bloomberg School of Public Health, Baltimore, Maryland;; 7 Medical Research Council Unit, Basse, The Gambia;; 8 Department of Paediatrics, University of Auckland, and; 9 Centre for International Health, University of Otago, Dunedin, New Zealand;; 10 Division of Infectious Disease and Tropical Pediatrics, Department of Pediatrics, Center for Vaccine Development, Institute of Global Health, University of Maryland School of Medicine, Baltimore; 11 Bill & Melinda Gates Foundation, Seattle, Washington;; 12 Medical Research Council, Respiratory and Meningeal Pathogens Research Unit, and; 13 Department of Science and Technology/National Research Foundation: Vaccine Preventable Diseases Unit, University of the Witwatersrand, Johannesburg, South Africa;; 14 Department of Pathology, University of Otago, and; 15 Microbiology Unit, Canterbury Health Laboratories, Christchurch, New Zealand;; 16 Department of Infectious Disease Epidemiology, London School of Hygiene & Tropical Medicine, United Kingdom;; 17 Center for Global Health and Development, Boston University School of Public Health, Massachusetts;; 18 Department of International Health, Center for Immunization Research, Johns Hopkins Bloomberg School of Public Health, Baltimore, Maryland; 19 Milken Institute School of Public Health, Department of Epidemiology and Biostatistics, George Washington University, Washington, District of Columbia; 20 Department of Biostatistics, Johns Hopkins Bloomberg School of Public Health, Baltimore, Maryland; and; 21 Division of Viral Diseases, National Center for Immunization and Respiratory Diseases, Centers for Disease Control and Prevention, Atlanta, Georgia; 22 Johns Hopkins Bloomberg School of Public Health, Baltimore, Maryland; 23 Bill & Melinda Gates Foundation, Seattle, Washington; 24 Centers for Disease Control and Prevention (CDC), Atlanta, Georgia; 25 The Emmes Corporation, Rockville, Maryland; 26 Nuffield Department of Clinical Medicine, University of Oxford, United Kingdom; 27 University of Otago, Christchurch, New Zealand; 28 ICDDR,b, Dhaka and Matlab, Bangladesh; 29 Medical Research Council, Basse, The Gambia; 30 KEMRI-Wellcome Trust Research Programme, Kilifi, Kenya; 31 Division of Infectious Disease and Tropical Pediatrics, Department of Pediatrics, Center for Vaccine Development, Institute of Global Health, University of Maryland School of Medicine, Baltimore, Maryland and Centre pour le Développement des Vaccins (CVD-Mali), Bamako, Mali; 32 Respiratory and Meningeal Pathogens Research Unit, University of the Witwatersrand, Johannesburg, South Africa; 33 Thailand Ministry of Public Health–US CDC Collaboration, Nonthaburi, Thailand; 34 Boston University School of Public Health, Boston, Massachusetts and University Teaching Hospital, Lusaka, Zambia

**Keywords:** PERCH, control selection, respiratory symptoms, pneumonia etiology, selection bias.

## Abstract

Many pneumonia etiology case-control studies exclude controls with respiratory illness from enrollment or analyses. Herein we argue that selecting controls regardless of respiratory symptoms provides the least biased estimates of pneumonia etiology. We review 3 reasons investigators may choose to exclude controls with respiratory symptoms in light of epidemiologic principles of control selection and present data from the Pneumonia Etiology Research for Child Health (PERCH) study where relevant to assess their validity. We conclude that exclusion of controls with respiratory symptoms will result in biased estimates of etiology. Randomly selected community controls, with or without respiratory symptoms, as long as they do not meet the criteria for case-defining pneumonia, are most representative of the general population from which cases arose and the least subject to selection bias.

One of the greatest challenges of case-control studies is identification of a suitable control group. If the control group is not representative of the population giving rise to the cases, results are susceptible to bias. For controls to be representative of the target population, they should be selected independently of the exposure of interest and must satisfy the conditions that (1) they could have become a case, and (2) if they had become a case, there is no a priori condition or circumstance that would have excluded them from detection or enrollment as a case. For studies of hospitalized pneumonia, the question of representativeness of controls usually centers on the choice of community vs hospital controls. Potential biases can be introduced by the selection of either hospital or community controls; some studies have chosen to enroll both groups of controls to hedge against each set of biases [1]. For the Pneumonia Etiology Research for Child Health (PERCH) study, we made an a priori decision to enroll controls only from the community because we believed this was the most representative sample introducing the least bias in evaluating both pneumonia etiology and risk factors, the primary objectives of PERCH; our rationale has been described previously [2].

Regardless of whether enrolling hospital or community controls, many case-control studies that evaluate risk factors for pneumonia or vaccine effectiveness do not restrict enrollment of controls based on the presence of respiratory symptoms [1, 3–10]. In contrast, many case-control studies of pneumonia etiology restrict the control group to asymptomatic or healthy children at the time of enrollment [11–15]. Some case-control studies have also excluded from analysis any controls who developed respiratory symptoms in the subsequent weeks following enrollment [11]. Only a few case-control studies have permitted children with respiratory illness to be included as controls [16–19]. When designing PERCH, we considered this question and decided that the optimal set of controls would include those without and with respiratory symptoms (excluding those with case-defining illnesses, namely severe and very severe pneumonia).

There are several reasons why investigators might choose to exclude controls with respiratory symptoms in pneumonia etiology studies. The first is to prevent misclassification of cases as controls. Most respiratory symptoms can be present in children whose illness severity ranges from upper respiratory tract infection (URTI) to very severe pneumonia. Drawing a line on that spectrum to firmly distinguish upper vs lower respiratory tract infections can lead to misclassification. A second reason for excluding controls with respiratory symptoms could be that, although controls may not meet all of the case-defining criteria at the time of screening, they may be in an intermediate state and develop case-defining pneumonia soon thereafter. Some investigators might consider that if these controls are already on the pathway to pneumonia, they should be thought of as “precases” and excluded from the control group. A third reason stems from the fact that diagnostic specimens from the site of primary infection (ie, the lung) are rarely obtained, so specimens from the upper respiratory tract are used to infer what is infecting the lung. Many of the same pathogens causing URTI (or colonization) can also cause pneumonia. It can be argued that controls with URTI will have elevated prevalence of some pathogens in the upper respiratory tract, and their inclusion as controls will underestimate the role of these pathogens in causing pneumonia in a case-control analysis.

In this article, we argue that these reasons do not justify the exclusion of controls with respiratory symptoms from pneumonia etiology studies such as PERCH. Where relevant, we present data from PERCH which support the decision to include all controls in the main PERCH etiology analysis.

## OVERVIEW OF THE PERCH STUDY

Identification and selection methods of cases and controls for the PERCH study have been described previously [2]. In brief, cases were hospitalized children aged 1–59 months with World Health Organization (WHO)–defined severe or very severe pneumonia enrolled at 9 sites in 7 countries: Bangladesh, The Gambia, Kenya, Mali, South Africa, Thailand, and Zambia. Exclusion criteria for cases were hospitalization within the previous 14 days, having been discharged as a PERCH case within the past 30 days, or resolution of lower chest wall indrawing following bronchodilator therapy for those with wheeze. Details about case definitions are provided elsewhere [20].

Controls were children aged 1–59 months living in the catchment area for cases; they were randomly selected from the community year round, and frequency matched to cases within the following age groups: 1 to <6 months, 6 to <12 months, 12 to <24 months, and 24 to <60 months. Potential controls were excluded only if they met the case definition of severe or very severe pneumonia, were hospitalized within the previous 14 days, or were discharged as a PERCH case within the past 30 days. The latter 2 criteria—also applied to cases to prevent (1) enrollment of nosocomial pneumonia cases and (2) reenrollment of cases for the same pneumonia episode—ensured that controls were representative of the population at risk of becoming a case.

Study staff trained on standardized clinical assessments examined potential case and control participants [21]. For controls, a clinical history of fever, cough, difficulty breathing, wheeze, inability to eat, runny nose, ear discharge, vomiting, diarrhea, abnormal sleepiness, and any other symptoms or signs reported by the guardian or study staff was collected. During clinical examination, children were assessed for the presence of cough, fever, rash, tachypnea, and the case-defining signs of severe and very severe pneumonia.

The institutional review board or ethical review committee approved the study protocol at each of the 7 institutions and at the Johns Hopkins Bloomberg School of Public Health. Parents or guardians of participants provided written informed consent.

## CONTROLS WITH RESPIRATORY SYMPTOMS IN PERCH

**Figure 1.**
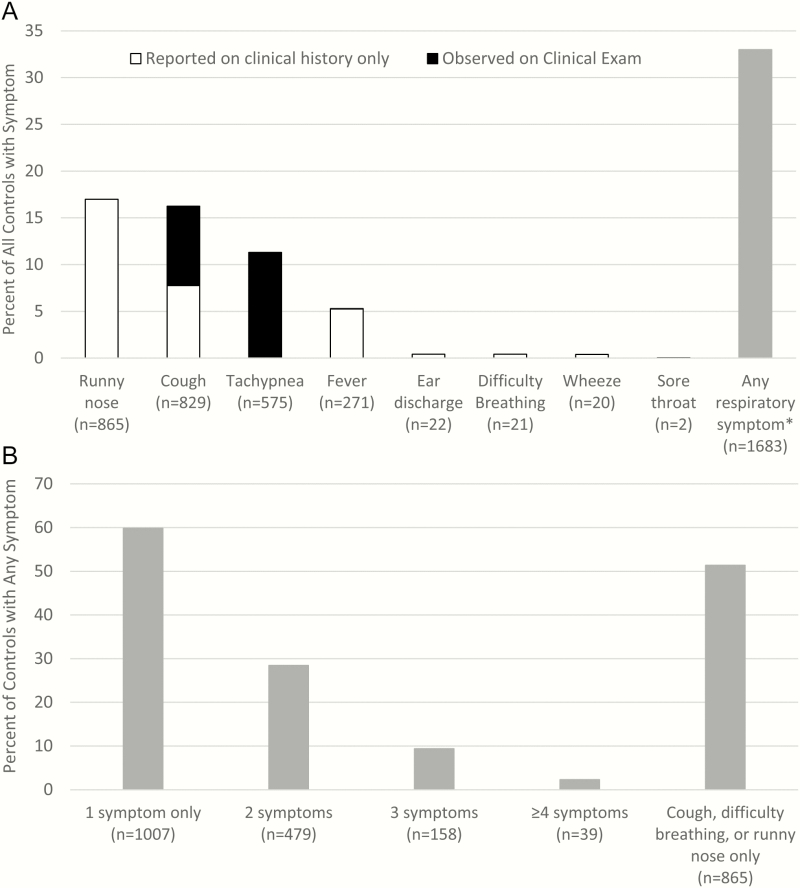
*A*, Distribution of respiratory symptoms among human immunodeficiency virus (HIV)–negative controls in the Pneumonia Etiology Research for Child Health (PERCH) Study. *Observed or reported. Clinical history recorded information on presence/absence of runny nose, cough, fever, ear discharge, difficulty breathing, wheeze, and sore throat as reported by guardian or study staff; clinical examination included assessment of cough, respiratory rate, and temperature by trained examiner. Tachypnea was defined as a respiratory rate ≥60 breaths per minute on clinical examination for children 1–2 months of age, ≥50 for children 2–11 months, and ≥40 for children 12–59 months. Fever on clinical examination was defined as temperature ≥38°C. *B*, Number of symptoms present among 1683 HIV-negative controls with any respiratory symptom. Respiratory symptoms (observed or reported) include runny nose, cough, tachypnea, fever, ear discharge, difficulty breathing, wheeze, and sore throat.

Symptoms of respiratory illness were common among controls in PERCH (Figure 1A). Among 5102 human immunodeficiency virus (HIV)–negative controls, 1683 (33.0%) had at least 1 respiratory symptom (runny nose, cough, tachypnea, difficulty breathing, wheeze, ear discharge, sore throat, or fever); the most common were runny nose (17.0%), cough (16.4%), tachypnea (11.3%), and fever (5.3%). Of those with respiratory symptoms, 676 (40.2%) had >1 symptom (Figure 1B). The prevalence of respiratory symptoms among controls varied by site; the percentage of controls with at least 1 symptom ranged from 14.5% in South Africa to 52.0% in Mali. To exclude controls with these common respiratory symptoms could lead to a large percentage of the general population of children being excluded as controls in some sites, calling into question the representativeness of the selected control population. Moreover, the variability of respiratory symptom prevalence among controls by site could lead to site differences in the representativeness of the control populations.

There is no standard definition of respiratory illness that can be easily applied to a control group. Should it be based on the presence of a single symptom or sign or require more than one, and if so which ones? Case-control studies of pneumonia have used various definitions of respiratory illness [11–19]. For example, tachypnea along with cough or difficulty breathing, is used to define nonsevere pneumonia in WHO clinical case management guidelines [22] because it is a sensitive measure that can be used to identify and treat the majority of children presenting with nonsevere pneumonia. Yet, tachypnea, which can also be caused by many other conditions, for example, fever alone (due to a nonrespiratory illness) or agitation, can have low specificity [23] and, thus, low positive predictive value for pneumonia, particularly among randomly selected children in the community where expected pneumonia prevalence is low. In the case of PERCH, 10.3% of controls without any other respiratory symptom had tachypnea. Therefore, defining the presence of respiratory illness in a population of controls with respiratory symptoms can be challenging.

## POTENTIAL MISCLASSIFICATION OF CASES AS CONTROLS

In pneumonia etiology studies, case definitions are often based on a constellation of symptoms, the identification of which relies on imperfect measurements. Because the symptoms of URTI and nonsevere pneumonia overlap with those of severe/very severe pneumonia, it is possible that children with respiratory symptoms enrolled as controls may have been cases who were missed due to errors in observation of case-defining symptoms and signs. Such differential misclassification can lead to results biased toward the null.

While it is possible that children with respiratory symptoms who were enrolled as controls did, in fact, have severe or very severe pneumonia, we believe this was a rare event in PERCH if it occurred. First, study staff at all sites underwent standardized training in the identification of PERCH cases and controls, as described elsewhere in this supplement [21]. Clinical standardization of health-worker personnel involved in study enrollment was achieved and maintained through on-site initial and refresher trainings, the development and use of a training website and training materials, and intermittent evaluations and competency assessments throughout the study. Every potential control underwent a standardized clinical examination to ensure they did not meet the case definition at the time of enrollment. Second, we sought evidence in the PERCH data for case-control misclassification. If controls with respiratory symptoms had truly been cases of severe or very severe pneumonia during enrollment, it is possible that they sought care at a hospital shortly thereafter. Four of the 9 study sites (Kilifi, Kenya; Sa Kaeo and Nakhon Phanom, Thailand; and Matlab, Bangladesh) were smaller rural sites where the study hospital served as the main referral hospital for the entire catchment area and where screening for PERCH occurred 24 hours a day, 7 days a week. Most cases of severe or very severe pneumonia seeking care would have presented to the study hospital and been identified by PERCH, though this may be less true of fatal cases at the Kenya site where a substantial number of child deaths occur outside of the main hospital at home or a closer health facility [24]. At these sites, we reviewed all enrolled PERCH cases to assess if they had been previously enrolled as a control. For all children enrolled both as a control and subsequently as a case, we reviewed time between enrollments to determine if the respiratory illness present during control enrollment was likely part of the same case-defining illness (ie, severe or very severe pneumonia). Of all 1942 controls enrolled at these 4 sites, 17 (0.9%) were subsequently enrolled as a PERCH case: 6 (0.3%) had at least 1 respiratory symptom or sign and 11 (0.6%) had no reported or observed respiratory symptoms or signs at the time of control enrollment. For the 6 controls with respiratory symptoms, the time between enrollment as a control and the reported first appearance of symptoms associated with the severe or very severe pneumonia episode ranged from 66 to 269 days (Table 1). For these children, sufficient time had passed between control and case enrollment to conclude that the illness present at control enrollment was not part of the same illness that resulted in later enrollment as a case.

**Table 1.** Characteristics of Pneumonia Etiology Research for Child Health (PERCH) Controls With Any Respiratory Symptom Subsequently Enrolled as PERCH Cases at 4 Study Sites—Kilifi, Kenya; Nakhon Phanom and Sa Kaeo^a^, Thailand; and Matlab, Bangladesh

Site	Age, mo	Symptoms Present at Control Enrollment	Days Between Enrollment as Control and as Case	Duration of Longest Symptom at Time of Case Enrollment, d	Days Between Control Symptoms and Case Symptoms	Chest Radiograph Result During Hospitalization as a Case^b^
Kilifi, Kenya	4	Cough, runny nose	223	14	209	Normal
Nakhon Phanom, Thailand	9	Rash^c^, tachypnea^c,d^	207	7	200	Abnormal
Nakhon Phanom, Thailand	5	Cough^c^, runny nose	271	2	269	Normal
Matlab, Bangladesh	16	Cough, tachypnea^c,d^	74	4	70	Uninterpretable
Matlab, Bangladesh	5	Cough	72	1	71	Abnormal
Matlab, Bangladesh	19	Cough^c^, runny nose	69	3	66	Abnormal

^a^No PERCH controls were later enrolled as cases in Sa Kaeo, Thailand.

^b^Abnormal chest radiograph result: presence of alveolar consolidation and/or other infiltrate.

^c^Symptom observed or measured by PERCH staff upon clinical examination at enrollment. All other symptoms reported by parent or guardian.

^d^ Tachypnea was defined as a respiratory rate ≥60 breaths per minute on clinical examination for children 1–2 months of age, ≥50 for children 2–11 months, and ≥40 for children 12–59 months.

We can also show in these 4 sites that the risk of subsequently developing severe or very severe pneumonia and being enrolled in PERCH was not different between those with and without respiratory symptoms. Among controls with at least 1 respiratory symptom (n = 758), 6 were subsequently enrolled as a PERCH case (0.8% risk), while among those with no symptoms (n = 1184), 11 were subsequently enrolled as a PERCH case (0.9% risk; relative risk, 0.9 [95% confidence interval {CI}, .3–2.3]). One would expect an excess risk of subsequent enrollment as a case among controls with respiratory symptoms if there were significant misclassification at enrollment among these controls.

We have less confidence that we would have detected misclassification of controls based on subsequent enrollment as PERCH cases in the more urban sites (ie, Bamako, Mali; Lusaka, Zambia; Soweto, South Africa; and Dhaka, Bangladesh). In these sites, hospitalizations for pneumonia might have occurred at clinical facilities other than the PERCH hospital. Additionally, controls could have presented later as cases during nonenrollment hours at the sites without 24/7 enrollment (Mali, South Africa, The Gambia). Despite these limitations, we found no evidence of controls with respiratory symptoms being later enrolled as a case at the study hospital, except at The Gambia site where 3 children were enrolled as cases 50, 81, and 230 days after being enrolled as a symptomatic control, again a sufficient amount of time to conclude that this was likely a separate illness.

We cannot claim with certainty that no controls with respiratory symptoms had severe or very severe pneumonia at the time of enrollment. In all sites, controls who subsequently developed severe pneumonia could have been hospitalized elsewhere. Moreover, in both urban and rural sites in low-income settings, children still die at home, so a control child who later developed fatal severe pneumonia could have been missed.

## UPPER RESPIRATORY TRACT INFECTION AS AN INTERMEDIATE CONDITION ON THE CAUSAL PATHWAY TO PNEUMONIA

Slightly different than the argument about misclassification (which could be summarized, “Was a control actually a case and we missed it?”) is the question about whether controls should be excluded if they have an intermediate condition for the outcome in question. For PERCH, this question can be rephrased as “If upper respiratory tract infection is on the causal chain or is an intermediate state on the path to severe or very severe pneumonia, then should controls with respiratory symptoms be excluded?” The similar risk of later being enrolled as a PERCH case observed for controls with vs without respiratory symptoms suggests that URTI is not on the causal pathway in pneumonia. However, in considering this question, we followed the epidemiologic rationale laid out by Charles Poole in his discussion of whether to exclude controls with intermediate states in case-control studies [25]. Poole argues that case-control studies are efficient means to mimic cohort studies and as such the controls need not be purged of persons in an intermediate state of the disease condition lest they deviate from the full cohort of persons from which the cases derived. The control group is only valid if it accurately reflects the exposure distribution among the population at risk for the outcome, regardless of intermediate states; a bias away from the null results when persons with the intermediate condition are excluded.

**Figure 2.**
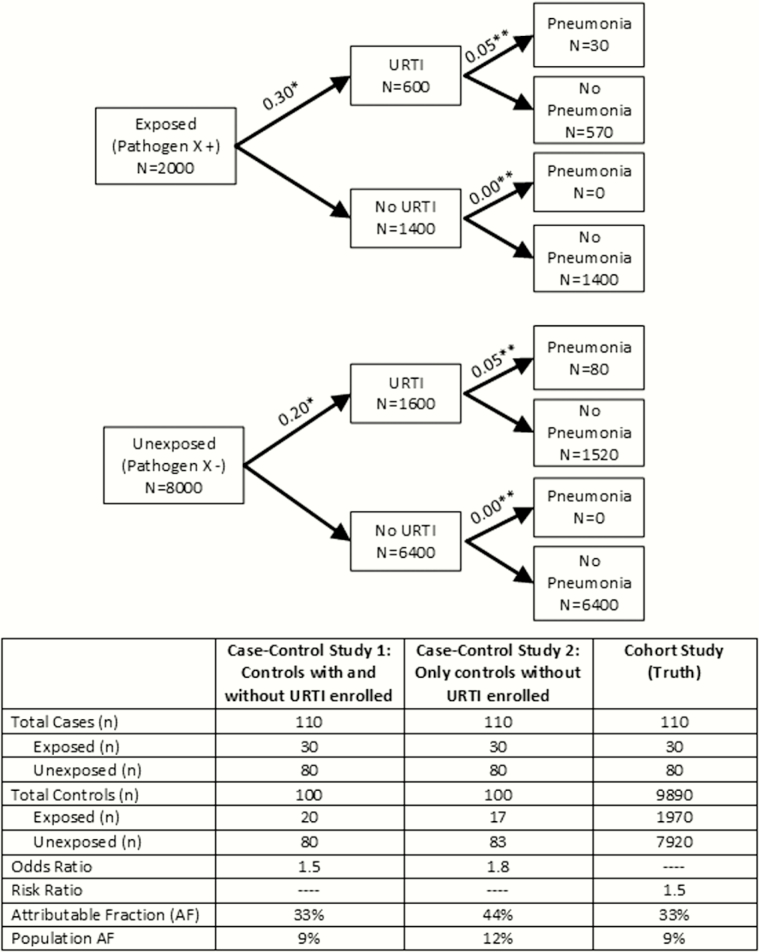
Depiction of realistic, hypothetical cohort in which pathogen X causes pneumonia solely through upper respiratory tract infection (URTI): comparison of a case-control study enrolling controls with and without URTI to a case-control study enrolling only controls without URTI. *The proportion of children that go on to develop URTI. **The proportion of children that go on to develop pneumonia.

A population of children in a low-income country will have a substantial prevalence of the intermediate condition of URTI, as shown for the PERCH sites, which, if excluded, will create an overestimation of the association between the exposure (ie, the pathogen in the nasopharynx) and the disease (ie, severe or very severe pneumonia). We adapted Poole’s figure of a hypothetical cohort in which the exposure causes disease only through an intermediate condition to illustrate the potential bias (Figure 2). Here, the exposure, pathogen X in the nasopharynx, causes pneumonia only through URTI. Pathogen X represents a highly prevalent pathogen in the community (20%), which increases the risk of pneumonia by 50% (risk ratio = 1.5) among those exposed to it. A case-control study that enrolls all cases and a random sample of 100 controls regardless of respiratory symptoms produces an odds ratio that accurately represents the true risk ratio. A case-control study that enrolls all cases, but excludes controls with respiratory symptoms and enrolls a random sample of healthy controls, overestimates the odds ratio by 20%, the attributable fraction by 33%, and the population attributable fraction by 33%. This theoretical phenomenon is illustrated by real-world data from a case-control pneumonia etiology study in Kenya in which the adjusted odds ratio for respiratory syncytial virus A infection was 12.5 (95% CI, 3.1–51.5) when the analysis was restricted to controls without respiratory symptoms, compared with 3.8 (95% CI, 2.2–6.6) when all controls were included in the analysis [16]. A recent meta-analysis of case-control studies of the etiology of acute lower respiratory infection also showed that when using only controls without respiratory symptoms, the attributable fraction in the exposed for most respiratory viruses was higher than when including all controls [14]. Of note, Poole also shows a scenario in which disease can occur among persons without the intermediate condition (ie, in our example, controls without URTI could develop pneumonia), which results numerically in a similar bias.

## CAN A PATHOGEN IN THE UPPER RESPIRATORY TRACT OF A CASE CAUSE RESPIRATORY ILLNESS UNRELATED TO THE PNEUMONIA EPISODE?

**Figure 3.**
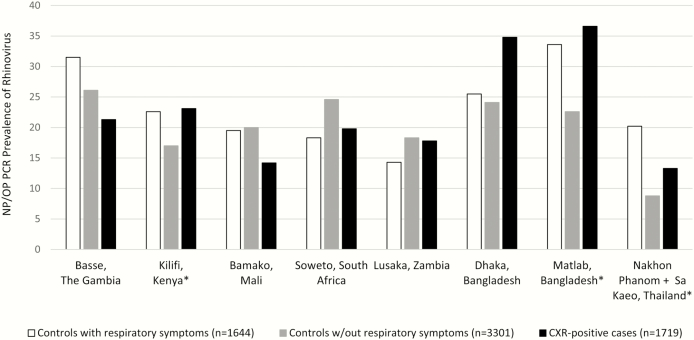
Rhinovirus nasopharyngeal/oropharyngeal (NP/OP) polymerase chain reaction (PCR) prevalence among chest radiograph (CXR)–positive cases and controls with and without symptoms of respiratory illness by site. CXR-positive was defined as the presence of alveolar consolidation and/or other infiltrate on CXR. Respiratory symptoms include runny nose, cough, tachypnea, fever, ear discharge, difficulty breathing, wheeze, and sore throat. *χ^2^ test comparing controls with vs without respiratory symptoms yielded *P* < .05.

Among patients with pneumonia, a pathogen detected in the nasopharynx/oropharynx might be the cause of the pneumonia, might be involved in the causal chain of pneumonia, or might represent a concurrent URTI unrelated to the pneumonic process. As to the first 2 possibilities, there is general acceptance that these can occur; however, there are few data to resolve whether the third scenario occurs. A case-control study like PERCH, which is cross-sectional in nature and has few specimens from the lung, is unable to answer this question definitively, and the progression from URTI to pneumonia is likely a complex interplay between pathogens and risk factors (eg, malnutrition, comorbid illnesses, smoke exposure) [26]. However, to exclude controls with respiratory symptoms would dictate that detection of these pathogens in pneumonia cases, beyond the prevalence observed in asymptomatic controls, would always be indicative of a causal role in pneumonia. This seems unlikely to be true for most pathogens detected in the upper respiratory tract of pneumonia cases. As an example from PERCH, we present the upper respiratory tract polymerase chain reaction (PCR) prevalence of rhinovirus, a virus thought to cause both URTI and, less commonly, pneumonia (Figure 3). Rhinovirus nasopharyngeal or oropharyngeal prevalence was higher in cases than controls in some sites and lower in cases than controls at other sites. However, rhinovirus had higher prevalence among controls with respiratory symptoms compared to asymptomatic controls at 6 of the 9 PERCH sites. The most notable difference was found at the Thailand sites where 20% of controls with respiratory symptoms tested positive for rhinovirus on nasopharyngeal or oropharyngeal PCR compared with 9% of asymptomatic controls. The outcome of a case-control analysis that included or excluded controls with respiratory symptoms would give very different results in Thailand. The high prevalence of rhinovirus in controls, both with and without respiratory symptoms, suggests that rhinovirus detection among cases constitutes a mixture of clinical states, including asymptomatic infection, URTI, and some in which rhinovirus is perhaps playing a causal role in pneumonia. Restricting the control group to children without respiratory symptoms would underestimate the prevalence of rhinovirus in the population from which the cases arose at most sites and overestimate the odds ratio of rhinovirus’ association with case status, consequently overestimating the etiology of rhinovirus as a cause of pneumonia.

Added to these biologic and interpretive reasons for inclusion of controls with upper respiratory symptoms are epidemiological principles that favor the inclusion of controls with respiratory symptoms. While the prevalence of exposures need not be similar in cases and controls, restrictions to subsets of the general population based on potential confounders or effect modifiers should be. For example, if the cases are restricted to girls, so should be the controls. Likewise, if the cases are not restricted in terms of respiratory symptoms, then the controls should not be either. Lack of such a restriction based on respiratory symptoms holds to the concept that case-control studies should mimic cohort studies as closely as possible, and one would not limit a cohort to only those children who do not develop respiratory illness.

## CONCLUSIONS

In summary, the PERCH study was designed to enroll controls, regardless of respiratory symptoms, as long as they did not meet the case definition of severe or very severe pneumonia. We have provided explanations and data to support this decision. We found no evidence to suggest that controls with respiratory symptoms were misclassified cases. We showed that as case-control studies are meant to mimic cohort studies, exclusion of controls with respiratory symptoms, some of whom might be in an intermediate state (ie, URTI) between health and disease (ie, severe and very severe pneumonia), can bias results away from the null. This decision also acknowledges that the presence of a pathogen in the upper respiratory tract of a pneumonia patient might be unrelated to the pneumonia episode. The sequence and synergy of multiple respiratory tract infections and their interplay with host factors in causing pneumonia is complicated, and a cross-sectional, case-control study like PERCH, regardless of which control group is used in the analysis, is not optimally designed to address this issue. By including all controls, regardless of respiratory symptoms, we aimed to uphold the epidemiological principle that the control group be representative of the target population from which cases arose to minimize bias in the case-control results.

## Notes

***Author contributions.*** M. M. H. performed analyses and drafted the initial manuscript. D. R. F., L. L. H., M. D. K., and K. L. O. provided significant guidance on the development of the manuscript. O. S. L., K. L. O., D. R. F., D. R. M., M. D. K., H. C. B., W. A. B., S. R. C. H., K. L. K., S. A. M., J. A. G. S., and D. M. T. conceived and designed the study and supervised study conduct. M. M. H., A. J. D., D. E. P., and C. P. were involved in study conduct. R. A. K. and S. Z. provided technical and analytic expertise. All authors reviewed and approved the manuscript. M. M. H. had full access to all the data in the study and had final responsibility for the decision to submit for publication.

***Acknowledgments.*** We acknowledge the significant contributions of the PERCH Study Group and all PERCH investigators. We offer our gratitude to the members of the Pneumonia Methods Working Group and PERCH Expert Group for their time and lending expertise to assist the PERCH Study Group. We offer sincere thanks to the patients and families who participated in this study.

***Pneumonia Methods Working Group.*** Robert E. Black, Zulfiqar A. Bhutta, Harry Campbell, Thomas Cherian, Derrick W. Crook, Menno D. de Jong, Scott F. Dowell, Stephen M. Graham, Keith P. Klugman, Claudio F. Lanata, Shabir A. Madhi, Paul Martin, James P. Nataro, Franco M. Piazza, Shamim A. Qazi, and Heather J. Zar.

***PERCH Expert Group.*** William C. Blackwelder, Harry Campbell, John A. Crump, Adegoke Falade, Menno D. de Jong, Claudio Lanata, Kim Mulholland, Shamim Qazi, Cynthia G. Whitney.

***Financial support.*** PERCH was supported by the Bill & Melinda Gates Foundation (grant number 48968 to the International Vaccine Access Center, Department of International Health, Johns Hopkins Bloomberg School of Public Health). J. A. G. S. was supported by a clinical fellowship from the Wellcome Trust of Great Britain (award number 098532).

***Supplement sponsorship.*** This article appears as part of the supplement “Pneumonia Etiology Research for Child Health (PERCH): Foundational Basis for the Primary Etiology Results,” sponsored by a grant from the Bill & Melinda Gates Foundation to the PERCH study of Johns Hopkins Bloomberg School of Public Health, Baltimore, Maryland.

***Potential conflicts of interest.*** M. D. K. has received funding for consultancies from Merck, Pfizer, Novartis, and grant funding from Merck. L. L. H. has received grant funding from Pfizer and GlaxoSmithKline. K. L. K. has received grant funding from Merck Sharp & Dohme. S. A. M. has received honoraria for serving on advisory boards for the Bill & Melinda Gates Foundation, Pfizer, Medimmune, and Novartis; has received institutional grants from GSK, Novartis, Pfizer, Minervax, and the Bill & Melinda Gates Foundation; and has served on speaker’s bureaus for Sanofi Pasteur and GSK. K. L. O. has received grant funding from Pfizer and GlaxoSmithKline and has served on technical advisory boards for Merck, Sanofi Pasteur, PATH, Affinivax, and ClearPath. All other authors report no potential conflicts. All authors have submitted the ICMJE Form for Disclosure of Potential Conflicts of Interest. Conflicts that the editors consider relevant to the content of the manuscript have been disclosed.
